# Neurorehabilitation in Transition: Neuroplasticity, Meaningful Outcomes, and Intelligent Technologies in the Highly Cited *Brain Sciences* Papers of 2024

**DOI:** 10.3390/brainsci16040376

**Published:** 2026-03-30

**Authors:** Rocco Salvatore Calabrò

**Affiliations:** Spinal Cord Unit, IRCCS Centro Neurolesi “Bonino-Pulejo”, 98124 Messina, Italy; roccos.calabro@irccsme.it

## 1. Introduction

Neurorehabilitation has entered a phase of rapid conceptual expansion. It is no longer understood only as the restoration of motor function after a focal lesion, nor as a late-stage adjunct to neurological care. It is increasingly recognized as a translational field that links biological recovery, behavioral adaptation, service organization, and digital innovation in order to improve function, participation, and long-term quality of life [1]. In this context, highly cited papers are imperfect but useful indicators of where the field is gaining intellectual and clinical traction. The 2024 highly cited papers selected from the Neurorehabilitation section of *Brain Sciences* reflect this. They include work on exercise-driven neuroplasticity in Parkinson’s disease (PD) and integrated rehabilitation pathways in spinal cord injury (SCI) (Contributions 1, 2). They also include analyses of clinically meaningful change in stroke trials and the underappreciated motor dimension of Alzheimer’s disease (AD) (Contributions 3, 4).

The selection is also notable for its breadth. Neurorehabilitation is represented here not only as motor retraining, but also as symptom decoding, cognitive and social reintegration, and technology-enabled decision support. The papers on social cognition in multiple sclerosis (MS) and on fatigue as a complex and difficult-to-quantify phenomenon illustrate that disability often emerges from domains that standard motor scales capture only partially (Contributions 5, 6). Read together, these contributions show a field moving away from narrow impairment models and toward a more layered understanding of recovery.

## 2. Recovery as a Biological, Behavioral, and Network Process

A central theme running through the selected papers is that rehabilitation is most effective when it is anchored in plausible mechanisms of brain and behavior change. Kaagman and colleagues review the effects of exercise on brain-derived neurotrophic factor (BDNF) levels and clinical outcomes in PD, linking physical training with a biologically credible model of neuroplasticity (Contribution 1). Their synthesis is important because it moves the conversation beyond the familiar claim that exercise is beneficial. It suggests instead that exercise may act on molecular pathways relevant to adaptive brain remodeling, a perspective that aligns with broader evidence on lifestyle-related modulators of plasticity in PD [2]. This is precisely the kind of mechanistic framing that strengthens rehabilitation science and makes intervention choices more defensible.

The review by Andrade-Guerrero and colleagues expands this logic into the neurodegenerative domain by focusing on motor impairments in AD (Contribution 4). This paper is particularly valuable because it resists the historical separation between cognitive neurology and rehabilitation medicine. Motor slowing, gait disturbance, postural instability, and reduced physical capacity are not merely secondary consequences of dementia. They are clinically meaningful features that influence autonomy, fall risk, caregiver burden, and therapeutic planning. By foregrounding these manifestations, the paper encourages a more integrated view of AD in which cognitive and motor trajectories are assessed together rather than in parallel.

An equally important network-based perspective emerges in the work of Tilton-Bolowsky and colleagues on post-stroke language recovery (Contribution 7). Their discussion of remapping and reconnecting the language system after stroke places aphasia rehabilitation within a contemporary framework of distributed brain networks rather than single-region substitution. This perspective is reinforced by the theoretical synthesis offered by Billot and Kiran, who detail how homeostatic and Hebbian mechanisms may shape post-stroke language recovery at the network level [3]. The convergence between these two papers is meaningful. It suggests that successful neurorehabilitation increasingly depends on understanding how residual circuits are engaged, modulated, and coordinated, not simply on whether an impaired function can be practiced more intensively.

## 3. What Neurorehabilitation Should Measure and Treat

If one major challenge is to understand how recovery occurs, another is to decide what counts as meaningful recovery in the first place. The review by Mishra and colleagues on minimal clinically important difference (MCID) values in stroke trials addresses this question directly (Contribution 3). Their contribution is timely because rehabilitation research often produces statistically significant results that are difficult to interpret at the bedside. By bringing attention to MCID, the paper reminds investigators and clinicians that numerical change is not enough. The decisive issue is whether an observed change is large enough to matter to patients, families, and care teams. In a field where interventions are often labor-intensive and resources are constrained, this distinction is crucial.

Maggio and colleagues offer a complementary perspective through their study of a multidisciplinary advanced rehabilitation pathway for SCI (Contribution 2). The importance of this paper lies not only in the outcomes reported, but also in the model of care it represents. Neurorehabilitation rarely succeeds through a single technique. It depends on coordinated action across physiotherapy, technological support, cognitive and psychological management, and sustained monitoring over time. The paper therefore highlights a point that is sometimes overshadowed by enthusiasm for novel devices. Organization of care is itself an intervention. When pathways are coherent, multidisciplinary, and responsive to complex disability, the probability of meaningful recovery increases.

Several selected papers further broaden the field by showing that relevant rehabilitation targets often sit outside conventional motor frameworks. Marafioti and colleagues examine social cognition deficits in MS and their relationship with quality of life (Contribution 6). This is a valuable reminder that successful rehabilitation must also address interpersonal functioning, emotional interpretation, and social participation. Rudroff’s review on fatigue adds another layer by emphasizing how persistent fatigue remains difficult to define, measure, and biologically parse, even though it often dominates patients’ lived experience (Contribution 5). His discussion of artificial intelligence (AI) as a possible tool for disentangling this complexity is especially relevant. It points toward a future in which rehabilitation phenotyping becomes more objective, multidimensional, and individualized.

## 4. Adjunctive Technologies and Physiologically Informed Interventions

The selected stroke-focused papers also show how contemporary neurorehabilitation is experimenting with interventions that aim to augment recovery rather than merely support compensation. Giorgi and colleagues review focal vibration therapy for motor deficits and spasticity in post-stroke rehabilitation (Contribution 8). The significance of this paper lies in its attention to a pragmatic clinical problem. Spasticity remains a major barrier to function, comfort, and training intensity. By assessing focal vibration as an adjunctive approach, the review addresses an area in which clinicians need realistic and scalable tools, especially when integrated with broader rehabilitation programs.

Meng and colleagues provide an equally useful overview of transcranial electrical stimulation (tES) for post-stroke motor recovery (Contribution 9). Their narrative review distinguishes among transcranial direct current stimulation, transcranial alternating current stimulation, and transcranial random noise stimulation, while also making clear that protocol heterogeneity remains a major limitation. This is an important message. Neuromodulation should not be treated as a generic enhancement technology. Its value depends on physiological rationale, patient selection, timing, and outcome alignment. The broader review by Saway and colleagues on neuromodulation for chronic stroke helps place this paper in a wider translational continuum that extends from non-invasive stimulation to vagus nerve stimulation, deep brain stimulation, and brain–computer interfaces [4]. Together, these contributions reflect a field that is becoming more ambitious but also more aware that ambition must be matched by mechanistic discipline.

## 5. From Immersive Rehabilitation to Intelligent Clinical Systems

A further theme emerging from the selected papers is that digital technologies are becoming meaningful only when they are tied to specific clinical questions. The systematic review by Maggio and colleagues on immersive virtual reality (VR) interventions in pediatric cerebral palsy is exemplary in this regard (Contribution 10). The paper does not treat VR as an inherently superior replacement for conventional therapy. Instead, it examines how immersive environments may enhance engagement, functional practice, and selected motor and cognitive outcomes within a rehabilitation program. That balanced framing is important because the field has moved beyond asking whether VR is exciting. The more relevant question is when immersive systems improve dose, motivation, ecological validity, or access in ways that standard therapy cannot. Recent broader work on immersive VR in neurological rehabilitation supports this direction while also underscoring the need for careful design, safety monitoring, and individualized implementation [5].

The two AI-centered papers selected for this editorial show a similar maturation. Al-Janabi and colleagues review current stroke solutions using AI, focusing on platforms that support rapid imaging interpretation and treatment decisions (Contribution 11). Although this work sits partly at the interface of acute neurology and rehabilitation, it matters to neurorehabilitation because earlier and more accurate characterization of injury shapes the downstream recovery pathway. Jeong and colleagues move even closer to rehabilitation workflow through their pilot study of an AI video analysis-based web application for diagnosing oropharyngeal dysphagia using videofluoroscopic swallowing study data (Contribution 12). This is a particularly concrete example of how AI can enter clinical practice. The value lies not in replacing specialist judgment, but in structuring complex visual information in a way that may improve consistency, efficiency, and therapeutic planning.

The broader literature suggests that these developments are not isolated signals. Recent reviews indicate that AI applications in adult stroke recovery and rehabilitation are already extending into movement analysis, prediction, robotics, and tailored intervention delivery [6]. The paper by Calderone and colleagues reaches a similar conclusion at the level of neurorehabilitation as a whole, arguing that AI may help personalize treatment, enhance monitoring, and expand access, while also raising questions of validation, ethics, and implementation [1]. These cautions matter. Technology in rehabilitation is most valuable when it clarifies clinical reasoning, strengthens patient-centered decision making, and increases the precision of care. It becomes less valuable when it creates complexity without improving relevance.

## 6. Conclusions

Taken together, the highly cited papers selected in the Neurorehabilitation section of *Brain Sciences* portray a field that is becoming more integrated, more mechanism-aware, and more clinically ambitious. They show that neurorehabilitation now extends from molecular and network models of plasticity to service pathways, symptom phenotyping, immersive environments, neuromodulation, and AI-assisted decision support. Just as importantly, they show that meaningful recovery cannot be reduced to a single scale or a single technology. It depends on choosing outcomes that matter, understanding the biology of adaptation, and embedding innovation within coherent models of care.

The strongest message emerging from this selection is that progress in neurorehabilitation will come from convergence. Exercise and broader lifestyle interventions, as suggested in PD-related work, may shape plasticity in ways that complement formal therapy (Contribution 11) and [2]. Network-based models of post-stroke language recovery may refine treatment targeting (Contribution 7) and [3]. Multidisciplinary pathways and sensitive outcome interpretation will remain essential (Contribution 2 and Contribution 3). Intelligent analytic tools may help translate promising interventions into durable functional benefit when they are embedded within those clinical structures (Contribution 11 and Contribution 12) and [6]. For that reason, the selected papers should not be seen as isolated achievements. They are better understood as markers of a field that is learning to connect mechanisms, measurement, and real-world care.

This is an encouraging direction for *Brain Sciences* and for neurorehabilitation more broadly. The next stage will require stronger trials, more precise phenotyping, better integration of biological and behavioral markers, and careful attention to implementation in everyday clinical settings. If these priorities are pursued with the same breadth represented by this highly cited collection, the field will be well positioned to deliver recovery that is not only measurable, but genuinely meaningful.

## Data Availability

Not applicable.
